# Supplementary material for: Pneumocystis jirovecii Pneumonia Diagnostic Approach: Real-Life Experience in a Tertiary Centre
Source: J Fungi (Basel). 2023 Mar 28;9(4):414. doi: 10.3390/jof9040414 (PMC10142180; doi:10.3390/jof9040414)
Supplement: Supplementary file 1 [file jof-09-00414-s001.zip › jof-2227896-supplementary.docx]

**Supplementary Materials**


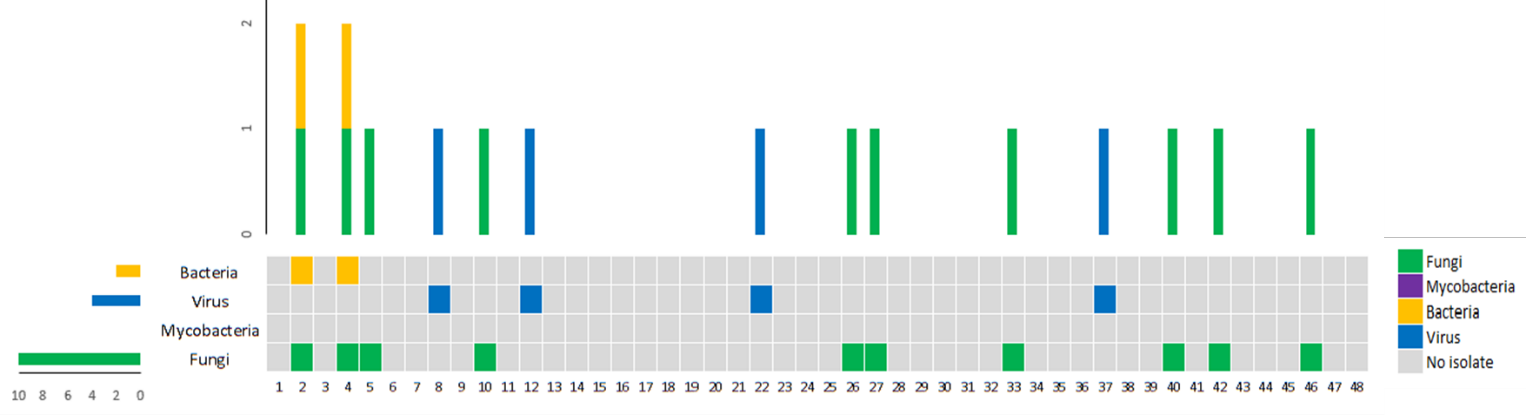


**Supplementary Figure 1:** Microorganisms isolated among 48 PJP cases. Fungi (on green) isolated were all *Candida* spp. Two cases recovered polymicrobial cultures (Case 2: *H. influenzae,* *S. pneumoniae*, *Candida* spp.; Case 4: *S. viridans* and *Candida* spp.). Viruses (on blue) detected were Cytomegalovirus.
